# Change in luminal diameter of the left internal thoracic artery anastomosed to the totally occluded left anterior descending coronary artery

**DOI:** 10.1186/s13019-016-0554-4

**Published:** 2016-11-28

**Authors:** Yochun Jung, Byoung Hee Ahn, Gwan Sic Kim, In Seok Jeong, Kyo Seon Lee, Sang Yun Song, Kook Joo Na, Sang Gi Oh

**Affiliations:** 1 Department of Thoracic and Cardiovascular Surgery, Chonnam National University Hospital, Chonnam National University School of Medicine, 42 Jebong-ro, Dong-gu Gwangju, 501-757 South Korea; 2 Department of Thoracic and Cardiovascular Surgery, Chonnam National University Hwasun Hospital, Chonnam National University School of Medicine, Hwasun, South Korea

**Keywords:** Coronary artery bypass grafting, Internal thoracic artery, Composite graft

## Abstract

**Background:**

Coronary artery bypass grafting (CABG) with a composite Y-graft made of the left internal thoracic artery (LITA) and another arterial graft has a risk for hypoperfusion. Changes over time in the diameter of the LITA anastomosed to the left anterior descending coronary artery (LAD) are not known.

**Methods:**

Data were collected for 71 patients who had undergone coronary angiography (CAG) immediately and at 1 year following off-pump CABG with a composite Y-graft made of the LITA and either the radial artery or the right gastroepiploic artery. These patients were divided into 2 groups depending on the degree of LAD stenosis. Group 1 (*n* = 28) consisted of patients with complete occlusion of the LAD. Group 2 (*n* = 43) consisted of patients with <90% stenosis of the LAD. The clinical state and luminal diameter of the LITA on immediate postoperative and postoperative 1-year CAG were compared and analyzed.

**Results:**

On the immediate postoperative CAG, mean LITA diameter of Group 1 was larger than that of Group 2 (2.09 ± 0.53 vs. 1.61 ± 0.33 mm, *P* = 0.01). Mean LITA diameter 1 year following CABG was also larger in Group 1 than in Group 2 (2.49 ± 0.31 vs. 2.10 ± 0.45 mm, *P* = 0.005). Both groups showed significant increases in the LITA diameters at postoperative 1 year.

**Conclusions:**

The LITA used as a composite Y-graft underwent remodeling, resulting in a larger diameter, to supply adequate myocardial blood. The degree of change in luminal diameter varied according to the severity of the LAD stenosis.

## Background

Since the publication of the study reporting that the left internal thoracic artery (LITA) anastomosed with the left anterior descending coronary artery (LAD) in coronary artery bypass grafting (CABG) showed excellent long-term patency [1], such anastomosis has been regarded as the gold standard in CABG. Furthermore, there is an increasing tendency to use total arterial revascularization (TAR), with the expectation that arterial grafts such as the radial artery (RA) or right gastroepiploic artery (RGEA) would have better long-term patency than that of a vein graft.

There are various strategies for TAR. Composite Y-grafting with the LITA and another arterial graft is one of the frequently-performed methods. TAR using a composite Y-graft, however, results in blood inflow from the LITA alone; thus, this method is controversial because of the risk for hypoperfusion [2].

This study included patients who underwent off-pump CABG by using a composite Y-graft with the LITA and the RA or the RGEA, and it aimed to investigate the adequacy of blood flow from the LITA to the LAD territory by using serial coronary angiographies (CAGs), which were performed just before the patients’ discharge from the hospital and 1 year after surgery for measurement of the diameter of the LITA right above the LAD anastomotic site.

## Methods

### Patients

Among 108 CABGs performed at our hospital between January 2000 and April 2005, 82 patients underwent off-pump TAR with a composite Y-graft. We excluded cases of; (1) poor distal run-off of the LAD due to diffuse atherosclerotic changes, (2) aneurysms of the left ventricle, (3) less than 20% left ventricular ejection fraction, (4) LAD stenosis of 90-100%, and (5) significant stenotic lesions on the LITA, and reviewed the medical records of the remaining 71 patients. The subjects were divided into 2 groups. Group 1 included cases of total occlusions of the LAD before surgery (*n* = 28, 4 women, mean age of 60.8 ± 8.9 years). Group 2, the control group, consisted of cases with <90% stenosis (*n* = 43 cases, 8 women, mean age of 58.9 ± 8.1 years). All patients underwent serial CAG, which were performed just before patients’ discharge from the hospital and 1 year after surgery. Changes in the luminal diameters of the LITA right above the LAD anastomotic site of both groups were compared. Before performing CAG, all the patients were informed of the necessity of the procedure and agreed to undergo it after fully understanding the possible complications. This study was approved by Chonnam National University Hospital’s Institutional Review Board, with patient consent waived.

### Surgical techniques

Electrocautery and hemoclip were used for skeletonized harvesting of the LITA, with the goal of complete cut-off up to the first branch. While harvesting the LITA, simultaneous harvesting of the other arterial grafts was performed. The RA was harvested from the non-dominant arm, and all patients underwent Allen’s Test before surgery. The approach to the RGEA following median sternotomy was carried out by extending an incision 2 to 4 cm toward the abdomen. After laparotomy, the RGEA was first verified by palpating it with the fingers. Then, the anterior serosa was separated by electrocautery, and the RGEA and vein were also separated. Warm and diluted papaverine solution (1 mg/mL) was sprayed onto the arterial graft externally to prevent vasospasm, without intraluminal injection. Harvested arterial grafts were wrapped in a gauze soaked with warm papaverine solution right up to its utilization. All arterial grafts were ligated after systemic heparinization (100 U/kg). During surgery, activated clotting time was maintained at 300 s or longer.

Surgeries were performed by a single surgeon (Ahn BH) and off-pump CABG using composite Y-graft was performed for all patients. Either 1) >70% of the segmental stenosis in the LAD or 2) >50% of diffuse stenosis in LAD originating from the LM was regarded as in indication of the composite Y-graft to LAD. On performing the coronary arterial anastomoses, an intracoronary shunt, a vascular sling, and carbon dioxide blower were used for securing the visual field at the incision area of the coronary arteries. The Guidant Acrobat^TM^ SUV Off-Pump System (Guidant Corporation, Santa Clara, California, USA) was used in the surgery. With respect to the composite Y-graft, the RGEA or the RA was anastomosed to the LITA at the area entering the pericardium. An 8-0 polypropylene suture was used for the anastomosis via the continuous running technique. After creating a Y-graft, the LITA was anastomosed to the LAD for all the patients, regardless of the state of collateral circulation. Subsequent anastomoses were performed in the following order: diagonal, obtuse marginal and right coronary branches. An intracoronary shunt was used for the performance of the LAD anastomosis, while a proximal vascular sling was used for the performance of the anastomoses of the remaining branches. In cases where the length of the RA was short for the target coronary artery anastomosis, the harvested RGEA was used as an extended graft. The RGEA and RA were anastomosed for cases of 70% or greater stenosis in the diagonal or left circumflex branches. Sequential anastomoses of the right coronary branches were performed for cases of 90% or greater stenosis. Anastomosis was performed for cases of coronary artery diameter of 1 mm or greater, with the aim of complete revascularization. An 8-0 polypropylene suture was used for anastomosis of the arterial graft. A diastolic dominant flow pattern was verified with a transit-time flowmeter (Transonic System Inc., NY, USA) after performing each anastomosis. At the end of each anastomosis, diluted papaverine solution was sprayed for the prevention of early vasospasm. Half of the calculated dosage of protamine injection was used for patients with tendencies of bleeding. In cases where bleeding through the thoracic drainage tube was thought to be insignificant at the intensive care unit after surgery, 100 mg of aspirin and 75 mg of clopidogrel were administered through a Levin tube.

### Quantitative angiographic evaluation

Angiography was performed before hospital discharge and 1 year after surgery, with mean durations of 13.3 ± 3.8 days and 12.7 ± 0.7 months, respectively. All the patients were medicated before CAG such that their systolic blood pressures were maintained at 110–140 mmHg. In order to reduce errors in angiography, values obtained from 2 different angles in the end diastolic phase were averaged. Angiographic findings were evaluated by 3 cardiovascular specialists. In cases of disagreement in opinions, decisions were made through discussion. Graft failure was defined as having an occlusion or stenosis rate of 70% or greater and included cases of a string sign, indicating diffuse stenosis.

### Statistical analysis

Continuous variables were presented as a mean ± standard deviation and were compared using the Student’s *t*-test or paired *t*-test as necessary. A *P* value <0.05 was considered statistically significant. The analyses were carried out using SPSS ver. 12.0 (SPSS Inc., Chicago, USA).

## Results

Clinical manifestations and preoperative echocardiography and CAG findings of both groups are as shown in Table 1. The total numbers of anastomoses for both Group 1 and Group 2 were 3.1 ± 0.8 and 2.97 ± 0.9, respectively and did not show any significant difference (*P* = 0.67). Numbers of anastomoses using the RGEA and the RA are tabulated in Table 2.

**Table 1:** Preoperative patients’ characteristics

Variables	Group 1	Group 2	*P* value
Number of Cases	28	43	
Sex (male : female)	24 : 4	35 : 8	
Age (years)	60.8 ± 8.9	58.9 ± 8.1	0.89
Preoperative diagnosis			
Stable angina	6	10	
Unstable angina	12	22	
Old myocardial infarction	2	1	
Acute myocardial infarction	8	10	
LV ejection fraction (%)	49.9 ± 14.7	57.5 ± 12.6	0.03
Infarct site on electrocardiogram			
LAD territory	7	9	
LCX territory	2	5	
RCA territory	10	16	
Coronary angiographic findings			
Left main disease	12	17	
Three-vessel disease	16	23	
Two-vessel disease	6	12	

*LV* left ventricle, *LAD* left anterior descending coronary artery, *LCX* left circumflex coronary artery, *RCA* right coronary artery

**Table 2:** Operative and postoperative data

Variables	Group 1	Group 2	*P* value
No. of anastomosis			
Mean	3.1 ± 0.8	2.97 ± 0.9	0.67
GEA	26	30	
RA	32	63	
Used grafts			
GEA	15	10	
RA	11	26	
GEA + RA	2	7	
Morbidity			
Bleeding reoperation	1	1	
Arrhythmia	4	5	
Wound infection	2	1	
Harvesting Related	0	0	

*No* number, *GEA* gastroepiploic artery, *RA* radial artery

The postoperative 1-year CAG revealed no case of a string sign in the LITA graft anastomosed to the LAD; however, 1 case of string sign was found in the RGEA graft anastomosed to the OM in Group 1 and 1 case in PDA-anastomosed RA in Group 2. Patency rates of the RGEA and RA immediately after surgery were 96.2% (25/26) and 100% (32/32), respectively, for Group 1, while patency rates of the RGEA and the RA immediately after surgery were 100% (30/30) and 100% (63/63), respectively, for Group 2. Patency rates of the RGEA and the RA 1 year after surgery were 96.2% (25/26) and 96.9% (31/32), respectively, for Group 1. Patency rates of the RGEA and the RA 1 year after surgery were 100% (30/30) and 96.8% (61/63), respectively, for Group 2. On follow-up CAG, the first branch of the LITA was not seen in any of the patients.

No in-hospital mortality was observed. One case from Group 2 developed low cardiac output syndrome in the immediate postoperative period but recovered. No cases of neurologic complications or myocardial ischemia or infarction were noted during the early postoperative and follow-up periods.

Significant increases in mean LITA diameters were found for both groups on the postoperative 1-year CAG. The diameters showed a 1.19-fold increase, from 2.09 ± 0.53 mm to 2.49 ± 0.31 mm, in Group 1, while they showed a 1.31-fold increase, from 1.61 ± 0.33 mm to 2.10 ± 0.45 mm, in Group 2. Mean LITA diameter of Group 1 was consistently larger than that of Group 2 on both immediate postoperative CAG and postoperative 1-year CAG (Table 3).

**Table 3:** Luminal diameter change of the left internal thoracic artery

	Group 1	Group 2	*P* value^a^
Immediate postoperative CAG	2.09 ± 0.53	1.61 ± 0.33	0.01
postoperative 1-year CAG	2.49 ± 0.31	2.10 ± 0.45	0.005
*P* value^b^	0.01	0.009	

*CAG* coronary angiography

^a^Group 1 v.s. Group 2

^b^Immediate postoperative v.s. postoperative 1-year CAG

One case in Group 1 was readmitted to the hospital for wound infection during the follow-up period. One case in Group 2 underwent percutaneous coronary intervention because of stenosis of the posterolateral branch anastomosis site. There was no cardiac-related death in either group.

## Discussion

As recent advances in percutaneous coronary intervention are associated with high success rates and reduced complications, CABG has largely become limited to patients with chronic diseases or severe multi-vessel coronary diseases [3]. Over time, patients tend to have a higher possibility of the need for a reoperation because of atherosclerotic changes in a graft rather than because of the native coronary disease itself. In order to reduce reoperation rates because of graft failure, it would be important to select a graft with good patency for primary CABG.

Owing to excellent long-term patency, arterial grafts have been increasingly used recently [4, 5]. In particular, the internal thoracic artery has the advantages of the low incidence of atherosclerosis, a functional arterial intima, an ideal vascular size that concurs with the coronary artery, and capacity for blood flow in accordance with changes in myocardial blood flow demand [1, 6]. For these reasons, anastomosis of the LITA to the LAD has been the basic surgical tenet of CABG.

Based on this fundamental procedure, TAR should be performed using various arterial grafts anastomosed with the LITA by using a composite Y- or T-graft configuration in cases requiring multiple grafting. A restrictive arterial graft could be utilized for TAR by using a composite graft, which has the advantage of a "no-touch technique" of the aorta. Nevertheless, it has the disadvantage of having the LITA as the only blood inflow source, and there are controversies regarding flow competition and inadequate myocardial blood flow in the immediate postoperative period [7, 8]. Thus, it is necessary to verify whether the composite LITA anastomosed to the LAD, which is the most important cardiac blood flow supply, could supply adequate blood volume.

Sakaguchi et al. asserted that a composite arterial Y-graft has less coronary flow reserve as opposed to that of independent grafts, and a small LITA size would call for precautions [2]. In contrast, Wendler et al. found that internal thoracic artery composite grafts could also provide adequate blood flow to the LAD [9]. Akasaka et al. insisted that an independent internal thoracic artery graft could supply adequate blood flow similar to that with saphenous vein grafts [10]. Markwirth et al. used a Doppler-guided wire to measure the flow of the proximal internal thoracic artery and coronary flow reserve after TAR through T-grafts, and observed that the functional and morphological adaptation capabilities of the internal thoracic artery were sufficient for higher flow volume requirements [11]. Citing the data measuring free flow in the operative field after formation of composite Y-graft with the LITA and the RA, Royces et al. reported that TAR with a composite conduit can also compensate for the myocardial blood flow requirement, as it shows a 2.3-fold reserve blood flow to the coronary vascular bed [12].

In patients subjected to TAR using the LITA as a composite graft, it is rare to find angiographic studies on whether sufficient blood flow could be supplied in the immediate postoperative period before achieving complete remodeling of the LITA anastomosed to the LAD. This study was based on patients with total occlusion of the LAD. The reasons were two-fold: (1) LITA flow to the LAD would not be affected by the degree of stenosis of the proximal LAD, and (2) LITA flow would be affected by myocardial blood flow demand alone.

The diameter of the LITA grafted to the LAD is closely related to the degree of proximal stenosis of the LAD, i.e. to the LAD flow volume via the LITA. The diameter of the LITA becomes larger when the coronary flow is LITA-dependent as a result of a severe proximal LAD stenosis. In contrast, the diameter of the LITA becomes smaller when the LAD flow is dependent on the native coronary artery because of a mild proximal LAD stenosis. Among children whose myocardial blood flow demand increases because of somatic growth, remodeling takes place along the length of the internal thoracic artery, as well as on its diameter [13]. That is, the internal thoracic artery graft is an active conduit with functional adaptability to myocardial flow demand, and not a passive conduit.

The remodeling of the internal thoracic artery is an integrated activity involving the endothelium, smooth muscle, fibroblasts, and extracellular matrix [14]. The internal thoracic artery is autoregulated to maintain the base shear stress of 15–20 dyne/cm^2^ [15]. Remodeling takes place on the basis of the Hagen-Poiseuille equation (τ = 4ησ/πγ3. τ = shear stress, η = blood viscosity, σ = blood flow, γ = graft diameter). In the early phase, the LITA flow would depend on flow velocity increase. However, as the high shear stress continues by means of high flow velocity, the diameter of the LITA increases, implicating a remodeling process in which the extent of shear force would decrease to base levels [16, 17].

Although the time when remodeling is completed has yet to be determined, Barner reported that the internal thoracic artery flow measured again 6–8 h after reoperation because of bleeding, showed a 40% increase, and that such increase implicated that flow adaptation of the ITA could occur immediately after surgery [18]. Akasaka et al. revealed that the flow velocity within 1 month of surgery was high and the flow velocity 1 year after surgery was low, while the internal thoracic artery diameter increased [10]. Such phenomenon could be analyzed by the Hagen-Poiseuille equation. Tagusari et al. reported that the LITA diameter significantly increased on angiography performed 14 days on the average after CABG using a composite Y-graft of the LITA and the RA, and reported that the LITA may undergo early adaptation [19]. However, the study did not report diameter change following the LAD stenosis or results of changes in the late period. Nakayama et al. suggested, from the results of angiography performed within 1 month after surgery and a mean interval of 4.5 ± 1.5 years after surgery, that the LITA diameter increased as the LAD stenosis became severe in cases without total LAD occlusion; however, they also reported that the LITA diameters measured for cases of total LAD occlusion were 2.27 ± 0.32 mm and 2.32 ± 0.38 mm, respectively, showing little change [6]. This suggests that LITA remodeling could be completed and may be able to supply sufficient blood flow required for the LAD territory within 1 month. In contrast, Akasaka et al. reported, from cases of independent ITA anastomosed with a totally occluded LAD, that the ITA diameters changed by flow demands, showing them to be 2.4 ± 0.1 mm, 1 month after surgery, while being 2.9 ± 0.2 mm 1 year after surgery [10].

In this study, both groups showed significant increases in the LITA diameters on postoperative 1-year CAG, although the mean LITA diameter of Group 1 was consistently larger than that of Group 2 in both immediate postoperative CAG and postoperative 1-year CAG. The increase in mean diameter of LITA in Group 2 seems to be related, in a certain part, to the increase in flow demand due to the progression of the LAD. Sanidas EA, et al. reported about the natural history of untreated nonculprit lesions in 697 patients with acute coronary syndrome. On angiographic study, 44 patients experienced substantial lesion progression (≥20% angiographic diameter stenosis increase) [20]. Even in a healthy man with a normal coronary angiogram, new coronary lesions do develop [21]. Therefore, it seems to be reasonable to assume that the progression of LAD stenosis has occurred and it might affect the diameter of LITA, although we have not checked the progression of LAD stenosis in the postoperative 1-year CAG in group 2. Interestingly, the mean LITA diameter of group 1 in the immediate postoperative CAG was not different to that of group 2 in the CAG performed 1 year after surgery (2.09 ± 0.53 vs. 2.10 ± 0.45 mm, *P* = 0.46). It shows that the dilatation of LITA, which happened over 1 year according to the progression of the LAD stenosis in group 2, had occurred in an immediately postoperative period in group 1, indicating that maximum possible enlargement of LITA can be achieved by the LITA-dependent blood flow in the immediate phase.

The increase in mean diameter of the LITA in Group 1 seems to show the remodeling of the LITA reflecting the increase in myocardial demand for blood flow during the first year. This might imply that maximum possible enlargement of the LITA in early postoperative period may not fully satisfy the myocardial demand in a certain circumstance. In this regard, the patients who underwent CABG to totally occluded LAD by using composite Y-graft had good to avoid excessive exercises that could increase myocardial demand during the early postoperative period, even though no myocardial ischemia-related complication was noticed in this study.

Limitations of this study are as follows: being an angiographic study, the flow reserve of the LITA, which is an early compensatory mechanism for high blood flow demand, could not be measured; possible effects of collateral flow of the coronary artery were not considered; the domain of myocardial infarction was not considered although preoperative echocardiography showed significant differences in left ventricular ejection fraction; the degree of change in native coronary artery disease was not taken into account in Group 2; the location of the anastomosis of the LITA and LAD was not considered; and the degree of remodeling immediately after surgery could not be investigated because the size of the LITA was not measured preoperatively. Further studies are needed to address these aspects and involve a larger number of patients with LAD stenosis of various levels.

## Conclusions

Changes in the diameters of the LITA on serial angiography, not considering functional LITA flow reserve, suggest that the LITA used as a composite graft during TAR undergoes remodeling, resulting in larger diameters, in order to provide adequate myocardial blood flow. The degree of change in luminal diameter varies according to the severity of the LAD stenosis.

## Abbreviations

CABG: Coronary artery bypass grafting
CAG: Coronary angiography
LAD: Left anterior descending coronary artery
LITA: Left internal thoracic artery
RA: Radial artery
RGEA: Right gastroepiploic artery
TAR: Total arterial revascularization
